# Development of a clinical nomogram for predicting hemorrhagic rupture in renal angiomyolipoma and analysis of molecular correlates (*CD34*, *PPARG*, and *PTEN*)

**DOI:** 10.1515/med-2026-1462

**Published:** 2026-06-05

**Authors:** Xiaobing Li, Jie Wan, Xuemei Li, Yao Zhang, Xianhui Hu

**Affiliations:** Science and Technology Industry Development Center, Chongqing Medical and Pharmaceutical College, Chongqing, China; College of Public Health, Chongqing Medical University, Chongqing, China; Special Medical Department, Daping Hospital, Army Medical University, Chongqing, China; Department of Urology, The First Affiliated Hospital of Chongqing Medical University, Chongqing, China; Urology Department, Chengdu Integrated TCM & Western Medicine Hospital, Chengdu, China

**Keywords:** renal angiomyolipoma (AML), hemorrhagic rupture, nomogram, *CD34*, *PPARG*, *PTEN*

## Abstract

**Objectives:**

Hemorrhagic rupture is a severe and potentially life-threatening complication of renal angiomyolipoma (AML). However, current clinical tools for accurately predicting hemorrhage risk remain limited. This study aimed to establish and validate a clinical nomogram for predicting hemorrhagic rupture in AML based on preoperative characteristics, and to investigate the underlying molecular correlates involving *CD34*, *PPARG*, and *PTEN* expression.

**Methods:**

A total of 379 patients with AML and 40 normal kidney tissue samples (2010–2021) were retrospectively analyzed. Patients were temporally divided into a training cohort (n=285) and an independent temporal validation cohort (n=94). Independent clinical predictors of hemorrhagic rupture were identified through univariate and multivariate logistic regression and incorporated into a nomogram. Model performance was evaluated using receiver operating characteristic (ROC) analysis and calibration plots in the validation cohort. Additionally, immunohistochemistry, qPCR, and ELISA were conducted to assess *CD34*, *PPARG*, and *PTEN* expression to explore biological mechanisms associated with rupture.

**Results:**

Tuberous sclerosis complex (TSC), tumor size ≥4 cm, rich vascular supply, and exophytic growth pattern were identified as independent predictors of hemorrhagic rupture (all p<0.05). The nomogram demonstrated excellent discrimination (AUC=0.967) and satisfactory calibration in the independent validation cohort. At the molecular level, immunohistochemical analysis revealed elevated *CD34* and *PPARG* expression and reduced *PTEN* expression in AML compared with normal tissues (p<0.05). Consistently, qPCR and ELISA assays confirmed significant upregulation of *CD34* and *PPARG* and downregulation of *PTEN* in hemorrhagic AML relative to non-hemorrhagic AML (p<0.001).

**Conclusions:**

The developed nomogram serves as a reliable pre-operative tool for individualized risk stratification of AML rupture. Furthermore, the aberrant expression of *CD34*, *PPARG*, and *PTEN* provides mechanistic insights into the vascular remodeling and fragility underlying AML hemorrhage, suggesting potential targets for future therapeutic research.

## Introduction

Renal angiomyolipoma (AML) is the most common benign renal neoplasm, accounting for approximately 3 % of all kidney tumors [1], [2], [3]. Clinically, AMLs are classified as either sporadic AML (SAML) or tuberous sclerosis complex–associated AML (TSC-AML) [4], 5], and pathologically into classic AML (CAML) and epithelioid AML (EAML) [6], 7]. Although generally benign, AMLs can lead to severe complications, among which intratumoral or retroperitoneal hemorrhage remains the most frequent and life-threatening event [8], [9], [10]. Hemorrhagic rupture of AML not only causes acute pain and hemodynamic instability but may also result in hypovolemic shock, posing significant risks to patient survival [11], 12].

The clinical management of AML is challenged by the unpredictable nature of hemorrhagic rupture. Given that AMLs are highly vascularized tumors, accurate prediction of hemorrhage risk is essential for guiding individualized treatment strategies [13], 14]. Previous studies have identified several clinical predictors-*e.g*., tumor size, vascularity, and the presence of tuberous sclerosis complex-but these parameters alone are insufficient to achieve robust risk stratification [15], [16], [17]. Consequently, the absence of a validated, quantitative predictive model limits the ability to balance timely intervention with overtreatment in patients with AML [18]. To date, existing management strategies-including active surveillance, selective embolization, surgical resection, and pharmacologic therapy with mTOR inhibitors-are largely guided by empirical experience rather than evidence-based predictive analytics [19]. Furthermore, while several clinical models have been proposed for other renal tumors, few have integrated molecular determinants to improve the accuracy of hemorrhage prediction in AML. This represents a crucial knowledge gap in the field [20], [21], [22].

Emerging evidence suggests that molecular dysregulation within tumor vasculature may underlie the susceptibility of AMLs to rupture [23]. In this context, endothelial and angiogenic markers such as *CD34*, peroxisome proliferator-activated receptor gamma (*PPARG*), and phosphatase and tensin homolog (*PTEN*) are of particular interest. *CD34*, a marker of vascular endothelial proliferation, reflects intratumoral angiogenesis and vascular fragility [24]. *PPARG*, a nuclear receptor involved in lipid metabolism and inflammation, has been implicated in tumor differentiation and vascular remodeling [25]. *PTEN*, a well-established tumor suppressor, regulates cell proliferation and angiogenic signaling pathways; its loss of expression may promote vascular instability and bleeding tendency [26]. Nevertheless, the relationship between these molecular markers and AML hemorrhagic behavior remains largely unexplored [27].

Given that current clinical parameters are insufficient for robust risk stratification of hemorrhagic rupture in renal AML, we hypothesized that the integration of clinicopathological factors with the molecular expression patterns of *CD34*, *PPARG*, and *PTEN* would significantly enhance predictive accuracy. Consequently, this study addressed the following research question: can a clinically applicable nomogram incorporating these clinical and molecular parameters reliably predict hemorrhagic rupture in a large patient cohort and provide novel insights into its pathogenesis?

## Materials and methods

### Patient cohort and study design

**Figure 1:**
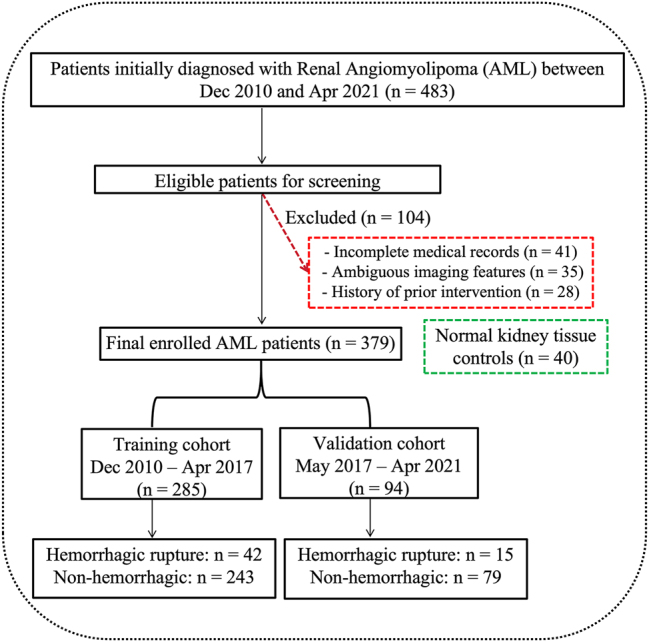
Flowchart of patient selection and study design. A total of 483 patients initially diagnosed with renal angiomyolipoma (AML) at The First Affiliated Hospital of Chongqing Medical University between December 2010 and April 2021 were screened. After exclusions (n=104), the final enrolled cohort of 379 patients was temporally divided into a training cohort (n=285) and an independent validation cohort (n=94). Additionally, 40 normal kidney tissue samples were included as controls for the subsequent molecular analysis.

This study was designed as a two-phase investigation comprising: (1) the development and validation of a clinical nomogram for predicting hemorrhagic rupture, and (2) the exploration of potential molecular mechanisms underlying tumor rupture. A total of 379 consecutive patients diagnosed with AML and 40 control individuals with normal kidney tissues were retrospectively enrolled from the Department of Urology, the First Affiliated Hospital of Chongqing Medical University (Chongqing, China) between December 2010 and April 2021. The study cohort comprised 120 males and 259 females. The mean age of the patients was 49.2 ± 12.8 years (range, 19–82 years). The diagnosis of AML was established based on contrast-enhanced computed tomography (CT) findings showing characteristic intratumoral adipose components. Tumor hemorrhage was strictly defined by the presence of perirenal or subcapsular hematoma confirmed on imaging. Patients with incomplete medical records, ambiguous imaging features, or a history of prior intervention were excluded. The detailed patient selection process and cohort division are illustrated in Figure 1. All patient data were retrieved from electronic medical records and physical archives. Specifically, archival biological samples (including FFPE tissue blocks and frozen serum) were retrieved for this study between October 2025 and December 2025.

### Data collection and variable definition

Comprehensive clinical and imaging data were collected for all enrolled patients. Variables included age, sex, hypertension, diabetes mellitus, pregnancy status, presence of tuberous sclerosis complex (TSC), extrarenal AMLs, renal cysts, tumor multiplicity, tumor laterality, presenting symptoms (asymptomatic vs. symptomatic, defined as flank pain, abdominal tenderness, hematuria, or hemorrhagic shock), tumor size, body mass index (BMI), polar position, tumor vascularity, and tumor growth pattern. Clinical symptoms were documented prior to any episode of AML rupture. Tumor size was measured as the maximum diameter on contrast-enhanced CT; for multiple lesions, the largest lesion was selected for analysis. Consistent with clinical management guidelines, tumor size was dichotomized as ≥4 cm or <4 cm. BMI was categorized based on Chinese population criteria (<24 kg/m^2^ vs. ≥24 kg/m^2^). Tumor vascularity was assessed using contrast-enhanced CT or color Doppler ultrasonography. A “rich blood supply” was defined by: (1) marked enhancement during the arterial phase on CT, (2) visualization of angiogenesis from ≥2 small arterial branches or one feeding artery, or (3) identification of ≥3 punctate blood flow signals on Doppler imaging. Lesions not meeting these criteria were classified as having a “poor blood supply.”

### Construction and validation of the nomogram

To ensure robust model evaluation, the 379 patients were temporally divided into two independent cohorts based on the time of diagnosis: a training cohort (n=285) comprising patients enrolled between December 2010 and April 2017, and an independent temporal validation cohort (n=94) comprising patients enrolled between May 2017 and April 2021. The training cohort was used to screen predictors and construct the nomogram, while the validation cohort was strictly reserved to evaluate the model’s predictive performance and generalizability. In the training cohort, univariate analysis was performed to compare baseline characteristics between ruptured and unruptured groups. Categorical variables were analyzed using the chi-square test or Fisher’s exact test, and continuous variables using the independent-samples t-test. Variables with p<0.05 in univariate analysis were entered into a multivariate logistic regression model. A backward stepwise elimination method was applied to identify independent risk factors for hemorrhagic rupture. Based on the multivariate regression coefficients (β-values), a nomogram was formulated using R software (version 4.2.1). The total risk score for each patient was calculated by summing the points of independent predictors. Model performance was assessed in the independent validation cohort using the following metrics: (1) Discrimination: evaluated using the area under the Receiver Operating Characteristic (ROC) curve (AUC). (2) Calibration: assessed using the Hosmer-Lemeshow goodness-of-fit test (p>0.05 indicates good fit) and visual inspection of calibration plots comparing predicted probabilities with observed outcomes.

### Immunohistochemistry (IHC) and scoring

Tissue specimens were fixed in 10 % neutral-buffered formalin, embedded in paraffin, and sectioned at 4 µm thickness. Antigen retrieval was performed in citrate buffer (pH 6.0) at 95–100 °C, followed by blocking with 10 % normal goat serum. Sections were incubated overnight at 4 °C with primary antibodies against *CD34* (cat. no. bs-0646R; BIOSS, Beijing, China), PPAR gamma (cat. no. bs-0530R; BIOSS, Beijing, China), and PTEN (cat. no. bs-0686R; BIOSS, Beijing, China) at a dilution of 1:50, followed by HRP-conjugated secondary antibodies. IHC evaluation was performed semi-quantitatively using the Remmele and Stegner Immunoreactive Score (IRS). Two experienced pathologists, blinded to clinical data and outcomes, independently evaluated the slides. Staining intensity (SI) was scored as: 0 (negative), 1 (weak), 2 (moderate), and 3 (strong). The percentage of positive cells (PP) was scored as: 0 (0–5 %), 1 (6–25 %), 2 (26–50 %), 3 (51–75 %), and 4 (>75 %). The final IRS was calculated by multiplying the intensity and percentage scores (IRS=SI × PP; range 0–12). To ensure reproducibility, any discrepancies in scoring between the two pathologists were resolved through discussion to reach a consensus. The IRS expression levels were dichotomized into “low expression” (IRS≤3) and “high expression” (IRS>3). This cutoff value was determined based on the median IRS of the study cohort to ensure balanced group sizes and statistical robustness for subsequent comparative analyses. Furthermore, this threshold is consistent with commonly utilized standards in oncological pathology research employing the Remmele and Stegner scoring system.

### qPCR analysis

Total RNA was extracted using TRIzol reagent (Invitrogen). RNA quality was verified (A260/A280 ratio 1.8–2.0), and cDNA was synthesized using the PrimeScript RT reagent kit (Takara). Quantitative PCR was performed on a 7500 Fast Real-Time PCR System using SYBR Green. To ensure statistical reliability, all qPCR reactions were performed in triplicate. Relative mRNA expression levels of *CD34*, *PPARG*, and *PTEN* were calculated using the 2ˆ−ΔΔCt method, with GAPDH used as the internal normalization control. Primer sequences are listed in Table 1.

**Table 1:** Primer sequences used for qPCR amplification of *CD34*, *PPARG*, and *PTEN*.

Gene	Forward primer (5′–3′)	Reverse primer (5′–3′)
*CD34*	CCT​CAG​TGT​CTA​CTG​CTG​GTC​T	GGA​ATA​GCT​CTG​GTG​GCT​TGC​A
*PPARG*	AGC​CTG​CGA​AAG​CCT​TTT​GGT​G	GGC​TTC​ACA​TTC​AGC​AAA​CCT​GG
*PTEN*	TGG​ATT​CGA​CTT​AGA​CTT​GAC​CT	GGT​GGG​TTA​TGG​TCT​TCA​AAA​GG
*GAPDH*	GGT​GAA​GGT​CGG​AGT​CAA​CG	CAA​AGT​TGT​CAT​GGA​TGA​CC

### Enzyme-linked immunosorbent assay (ELISA)

Serum concentrations of *CD34*, *PPARG*, and *PTEN* were measured using commercial ELISA kits (Cloud-Clone Corp., Wuhan, China; cat. no. SEB959Hu for *CD34*, SEA886Hu for *PPARG*, and SEF822Hu for *PTEN*) strictly following the manufacturer’s instructions. Optical density was measured at 450 nm, and concentrations were derived from standard curves. All samples were analyzed in duplicate to ensure assay reproducibility.

### Statistical analysis

Statistical analyses were performed using R software (version 4.2.1) and SPSS version 25.0. Continuous data are presented as mean ± standard deviation (SD) and categorical data as frequencies (percentages). Group comparisons were performed using Student’s t-test, Chi-square test, or Fisher’s exact test. For molecular analysis, differences between multiple groups were analyzed using one-way ANOVA followed by Tukey’s post hoc test. Pearson’s correlation coefficient was used to assess the relationship between *CD34* and *PPARG* expression. Specifically, Logistic regression was employed as the primary analytical tool for risk prediction instead of a time-to-event model (*e.g*., Cox proportional hazards regression). This choice was necessitated by our study design, which functioned as a cross-sectional/case-control investigation. A significant proportion of patients in the rupture group presented with acute symptoms or spontaneous hemorrhage as their primary complaint at initial admission, meaning there was no identifiable “unruptured observation period” or longitudinal follow-up from initial diagnosis to the event of rupture. Therefore, we modeled the clinical status of “hemorrhagic rupture at presentation” as a binary outcome. A two-sided p-value <0.05 was considered statistically significant.

### Research ethics

The study was conducted in accordance with the Declaration of Helsinki (as revised in 2013). The study protocol was reviewed and approved by the Institutional Review Board (IRB) of Chongqing Medical and Pharmaceutical College (Approval No. KYLLSC20251020011).

### Informed consent

Informed consent was obtained from all individuals included in this study, and samples were stored for future scientific research and teaching purposes according to the standard operating procedures of the hospital.

## Results

### Patient characteristics and cohort definition

A total of 379 patients with a confirmed diagnosis of AML were enrolled. To facilitate robust model validation, the study population was temporally divided based on the time of diagnosis into a training cohort (n=285, enrolled 2010–2017) and an independent temporal validation cohort (n=94, enrolled 2017–2021). As summarized in Table 2, the training and validation cohorts were well-balanced. No statistically significant differences were observed regarding major clinical and radiological parameters, including age, sex, tumor size, hypertension, diabetes, renal cysts, tumor multiplicity, laterality, growth pattern, BMI, polar location, extrarenal AML involvement, and tumor vascularity (all p>0.05). This comparable baseline distribution confirms that the temporal validation cohort is representative of the target population, providing a reliable basis for testing the model’s generalizability.

**Table 2:** Comparison of clinical features between the training and validation sets.

Clinical feature	All patients (n=379)	Training set (n=285)	Validation set (n=94)	*χ*²/T	p-Value
Age (years)	49.2 ± 12.77	48.3 ± 12.92	–	0.629	0.53
Sex				0.004	0.952
Female	259 (68.34 %)	195 (68.42 %)	64 (68.09 %)		
Male	120 (31.66 %)	90 (31.58 %)	30 (31.91 %)		
Tuberous sclerosis complex				0.184	0.668
No	351 (92.61 %)	263 (92.28 %)	88 (93.62 %)		
Yes	28 (7.39 %)	22 (7.72 %)	6 (6.38 %)		
Hypertension				1.357	0.244
No	317 (83.64 %)	242 (84.91 %)	75 (79.79 %)		
Yes	62 (16.36 %)	43 (15.09 %)	19 (20.21 %)		
Diabetes				1.177	0.278
No	359 (94.72 %)	272 (95.44 %)	87 (92.55 %)		
Yes	20 (5.28 %)	13 (4.56 %)	7 (7.45 %)		
Other organs containing AMLs				0.506	0.477
No	322 (84.96 %)	240 (84.21 %)	82 (87.23 %)		
Yes	57 (15.04 %)	45 (15.79 %)	12 (12.77 %)		
Renal cyst				0.988	0.32
No	288 (75.99 %)	213 (74.74 %)	75 (79.79 %)		
Yes	91 (24.01 %)	72 (25.26 %)	19 (20.21 %)		
Multiplicity				0.201	0.654
Single	320 (84.43 %)	242 (84.91 %)	78 (82.98 %)		
Multiple/bilateral	59 (15.57 %)	43 (15.09 %)	16 (17.02 %)		
Tumor location				−1.19	0.234
Right	173 (45.65 %)	124 (43.51 %)	49 (52.13 %)		
Left	158 (41.69 %)	125 (43.86 %)	33 (35.11 %)		
Bilateral	48 (12.66 %)	36 (12.63 %)	12 (12.77 %)		
Growth pattern				0.142	0.707
<50 % exophytic growth	139 (36.68 %)	103 (36.14 %)	36 (38.30 %)		
≥50 % exophytic growth	240 (63.32 %)	182 (63.86 %)	58 (61.70 %)		
Polar position				−0.826	0.409
Others	37 (9.76 %)	29 (10.18 %)	8 (8.51 %)		
Upper	109 (28.76 %)	85 (29.82 %)	24 (25.53 %)		
Lower	129 (34.04 %)	94 (32.98 %)	35 (37.23 %)		
Middle	104 (27.44 %)	77 (27.02 %)	27 (28.72 %)		
Tumor blood supply				0.526	0.468
Poor	234 (61.74 %)	173 (60.70 %)	61 (64.90 %)		
Rich	145 (38.26 %)	112 (39.30 %)	33 (35.09 %)		
Symptoms at presentation				0.051	0.82
Asymptomatic	287 (75.73 %)	215 (75.44 %)	72 (76.60 %)		
Symptomatic	92 (24.27 %)	70 (24.56 %)	22 (23.40 %)		
Tumor size				1.21	0.271
<4 cm	220 (58.05 %)	170 (59.65 %)	50 (53.19 %)		
≥4 cm	159 (41.95 %)	115 (40.35 %)	44 (46.81 %)		
BMI				−0.232	0.816
Normal	224 (59.10 %)	167 (58.60 %)	57 (60.64 %)		
Lean	7 (1.85 %)	4 (1.40 %)	3 (3.19 %)		
Overweight	120 (31.66 %)	95 (33.33 %)	25 (26.60 %)		
Obese	28 (7.39 %)	19 (6.67 %)	9 (9.57 %)		

### Radiological features of hemorrhagic AML

**Figure 2:**
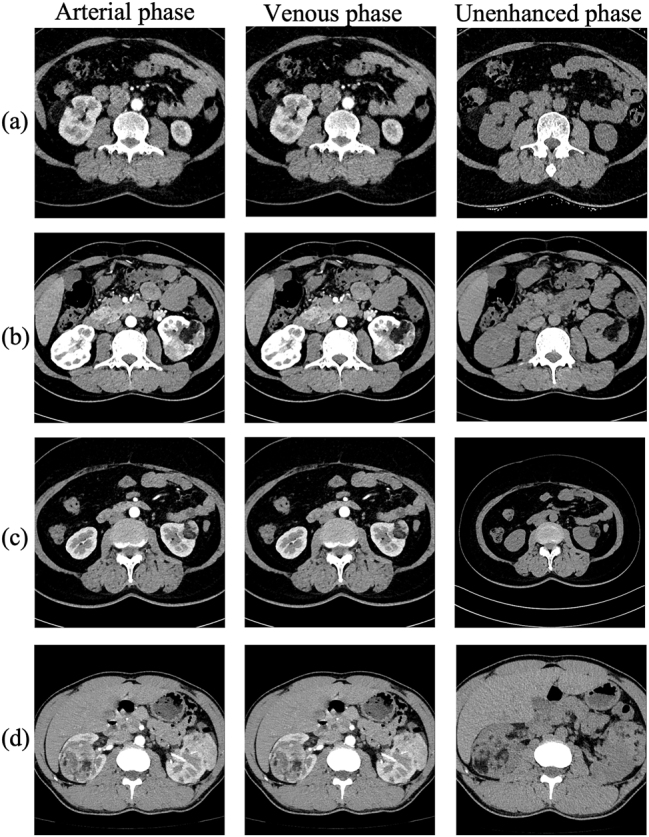
Representative contrast-enhanced CT images of non-hemorrhagic AMLs. Axial CT images from four different patients with non-hemorrhagic renal angiomyolipoma (panels a–d). For each patient, representative slices from the unenhanced, corticomedullary (arterial), and nephrographic (venous/contrast-enhanced) phases are shown. (a) A small, well-defined cortical AML showing uniform intratumoral fat attenuation without hemorrhage. (b) A medium-sized exophytic AML exhibiting heterogeneous enhancement and clear margins. (c) A fat-poor AML demonstrating mild enhancement and no perirenal extension. (d) A large mixed-density AML with internal vessels but intact capsule and no perirenal hematoma. All cases illustrate typical imaging hallmarks of non-ruptured AMLs, characterized by preserved contour, contained enhancement, and absence of hemorrhagic features.

**Figure 3:**
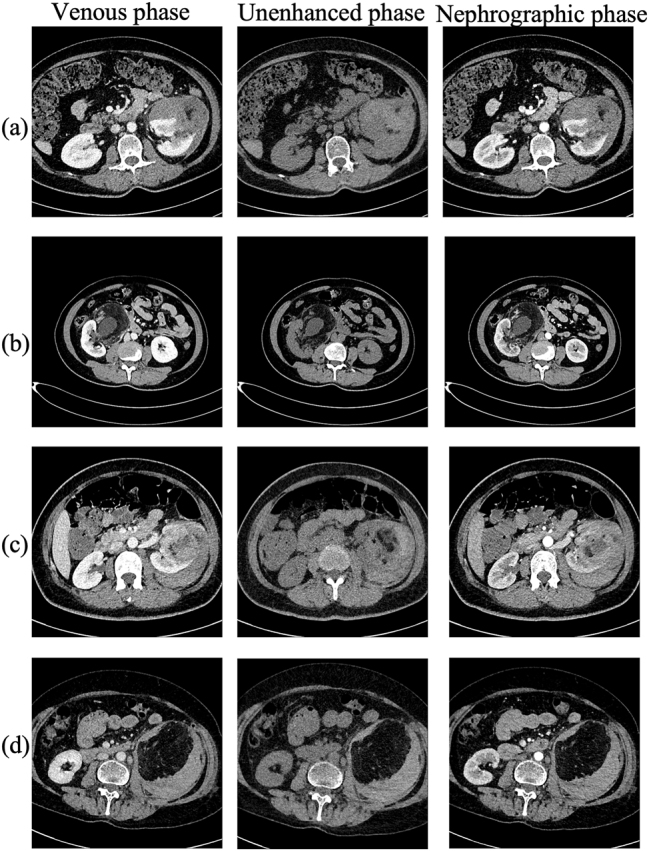
Representative contrast-enhanced CT images of hemorrhagic AMLs with capsular rupture. Axial CT images from four different patients with hemorrhagic renal angiomyolipoma (panels a–d). For each patient, representative slices from the unenhanced, corticomedullary (arterial), and nephrographic (venous/contrast-enhanced) phases are presented. (a) A ruptured AML showing high-attenuation intratumoral hemorrhage and perirenal hematoma. (b) A large exophytic AML with capsular disruption and active contrast extravasation during the enhancement phase. (c) A fat-poor AML with mixed intratumoral densities and subcapsular bleeding. (d) A posteriorly located AML demonstrating heterogeneous enhancement and perirenal fluid collection extending along Gerota’s fascia. These cases highlight the phase-dependent CT manifestations of hemorrhagic rupture, including intratumoral bleeding, capsular breach, and perirenal hematoma formation.

Figure 2 presents representative multiphasic CT scans of non-hemorrhagic AMLs, demonstrating characteristic intratumoral fat attenuation, heterogeneous enhancement, and preserved renal contour. In contrast, Figure 3 displays the distinct features of hemorrhagic AML rupture, characterized by intratumoral hyperattenuation, capsular disruption, active contrast extravasation, and perirenal hematoma formation. These distinct phase-dependent enhancement patterns and morphologic hallmarks served as the radiologic foundation for variable selection in the predictive model.

### Univariate and multivariate analysis of risk factors

In the training cohort, hemorrhagic rupture occurred in 42 of 285 patients (14.7 %). Univariate logistic regression analysis identified four parameters significantly associated with AML rupture: presence of TSC (p<0.001), exophytic tumor growth pattern (p=0.004), rich tumor vascularity (p<0.001), and tumor size ≥4 cm (p<0.001). Other clinical characteristics-including sex, hypertension, diabetes mellitus, renal cysts, tumor multiplicity, laterality, BMI, and polar location-were not significantly associated with rupture (all p>0.05) (Table 3). Multivariate logistic regression analysis confirmed that TSC, tumor size ≥4 cm, rich tumor vascularity, and exophytic tumor growth pattern were independent predictors of hemorrhagic rupture (Table 4). Demographic factors such as age and sex did not demonstrate independent predictive value. These findings underscore that intrinsic tumor biology (TSC status, vascularity, growth dynamics) and tumor burden (size) are the primary determinants of hemorrhagic instability.

**Table 3:** Univariate analysis of clinical factors in the training set.

Clinical feature	Training set (n=285)	Without hemorrhage (n=243)	With hemorrhage (n=42)	χ^2^/T/Z	p-Value
Age (years)	48.4 ± 12.42	–	47.3 ± 15.59	0.51	0.611
Sex				1.805	0.179
Female	195 (68.42 %)	170	25		
Male	90 (31.58 %)	73	17		
Tuberous sclerosis complex				54.195	<0.001
No	263 (92.28 %)	236	27		
Yes	22 (7.72 %)	7	15		
Hypertension				0.005	0.946
No	242 (84.91 %)	209	33		
Yes	43 (15.09 %)	34	9		
Diabetes				2.383	0.123
No	272 (95.44 %)	232	40		
Yes	13 (4.56 %)	11	2		
Other organs containing AMLs				0.055	0.814
No	240 (84.21 %)	208	32		
Yes	45 (15.79 %)	35	10		
Renal cyst				0.603	0.437
No	213 (74.74 %)	181	32		
Yes	72 (25.26 %)	62	10		
Multiplicity				1.948	0.377
Single	242 (84.91 %)	208	34		
Multiple/Bilateral	43 (15.09 %)	35	8		
Tumor location				−1.112	0.266
Right	124 (43.51 %)	108	16		
Left	125 (43.86 %)	107	18		
Bilateral	36 (12.63 %)	28	8		
Growth pattern				8.094	0.004
<50 % exophytic growth	103 (36.14 %)	96	7		
≥50 % exophytic growth	182 (63.86 %)	147	35		
Polar position				−0.042	0.966
Others	29 (10.18 %)	22	7		
Upper	85 (29.82 %)	78	7		
Lower	94 (32.98 %)	76	18		
Middle	77 (27.02 %)	67	10		
Tumor blood supply				21.318	<0.001
Poor	173 (60.70 %)	161	12		
Rich	112 (39.30 %)	82	30		
Symptoms at presentation				77.551	<0.001
Asymptomatic	215 (75.44 %)	206	9		
Symptomatic	70 (24.56 %)	37	33		
Tumor size				51.422	<0.001
<4 cm	170 (59.65 %)	166	4		
≥4 cm	115 (40.35 %)	77	38		
BMI				−1.607	0.108
Normal	167 (58.60 %)	148	19		
Lean	4 (1.40 %)	3	1		
Overweight	95 (33.33 %)	75	20		
Obese	19 (6.67 %)	17	2		

**Table 4:** Multivariate stepwise logistic regression analysis of clinical factors in the training set.

Clinical factor	β	SE	WALD	p-Value	OR	95 % CI
Tuberous sclerosis complex	2.572	0.719	12.804	<0.001	13.098	3.201–53.601
Symptoms at presentation	1.188	0.485	6.004	0.014	3.279	1.268–8.479
Tumor size	2.114	0.481	19.294	<0.001	8.283	3.225–21.277
Tumor blood supply	2.344	0.634	13.695	<0.001	10.428	3.013–36.096
Constant	−5.238	0.685	58.54	<0.001	0.005	–

### Construction and validation of the nomogram

**Figure 4:**
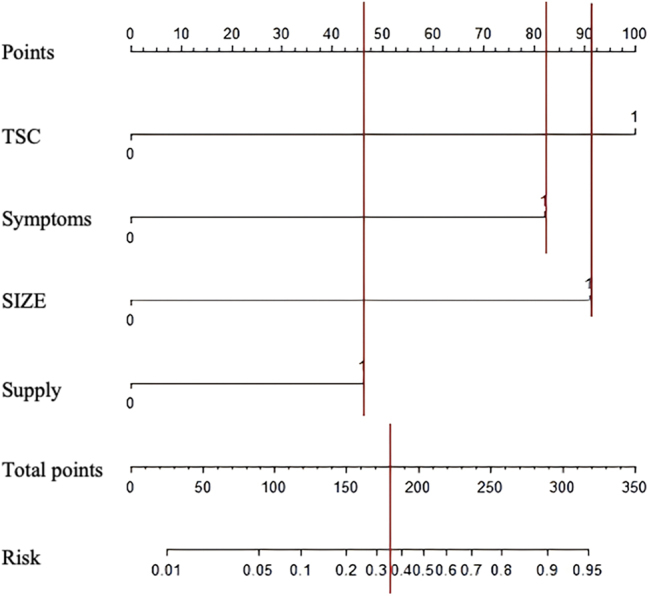
Nomogram for predicting the risk of hemorrhagic rupture in renal angiomyolipoma. The nomogram was constructed based on four independent predictors: TSC, tumor size, tumor blood supply, and symptomatic presentation. Each variable corresponds to a specific point value according to its contribution to rupture risk, with TSC, tumor diameter ≥4 cm, symptomatic presentation, and rich tumor blood supply assigned 100, 90, 82.5, and 45 points, respectively. The total score, obtained by summing individual points, corresponds to a predicted probability of hemorrhagic rupture, as shown on the bottom scale. For example, a patient with TSC (100 points), symptomatic presentation (82.5 points), tumor size <4 cm (0 points), and poor vascularity (0 points) achieves a total score of 182.5, corresponding to an estimated rupture probability of approximately 35 %.

**Figure 5:**
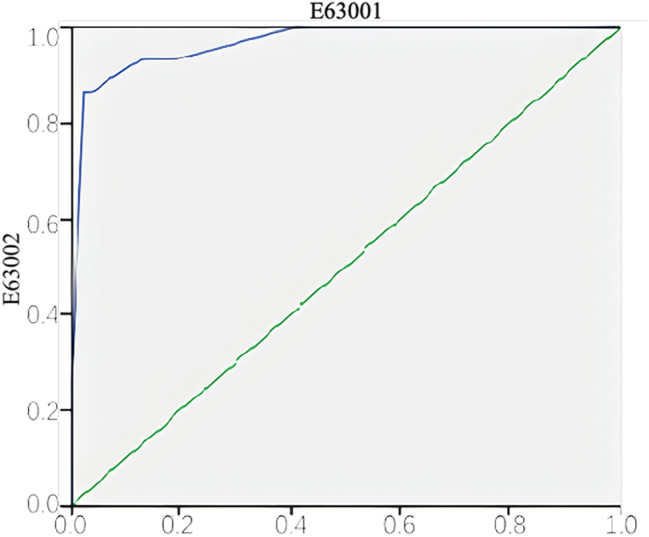
ROC curve of the nomogram predicting hemorrhagic rupture in renal angiomyolipoma. The discriminative performance of the nomogram was assessed in the validation cohort (n=94), in which 15 patients (15.95 %) experienced hemorrhagic rupture. The area under the ROC curve (AUC) was 0.967 (95 % CI: 0.912–0.993), indicating excellent predictive accuracy. The Hosmer-Lemeshow goodness-of-fit test (χ^2^=2.982, p=0.561) demonstrated satisfactory model calibration.

Based on the multivariate analysis, a quantitative nomogram was established incorporating the four independent predictors. Weighted scores were assigned to each variable: TSC (100 points), tumor size ≥4 cm (90 points), exophytic growth pattern (82.5 points), and rich vascularity (45 points), yielding a maximum total score of 317.5. Figure 4 illustrates the nomogram, enabling individualized risk stratification. For instance, a patient with TSC and an exophytic tumor, but with a size <4 cm and poor vascularity, would have a total score of 182.5, corresponding to an estimated rupture probability of approximately 35 %. The model’s performance was rigorously tested in the independent temporal validation cohort (n=94), where 15 patients (15.95 %) experienced rupture. The model achieved excellent discrimination with an AUC of 0.967 (Figure 5). Furthermore, the Hosmer-Lemeshow goodness-of-fit test demonstrated satisfactory calibration (χ^2^=2.982, p=0.561), indicating a high degree of agreement between the predicted probabilities and the actual observed rupture rates in the validation set.

### Molecular correlates: immunohistochemical expression of *CD34*, *PPARG*, and *PTEN*

**Figure 6:**
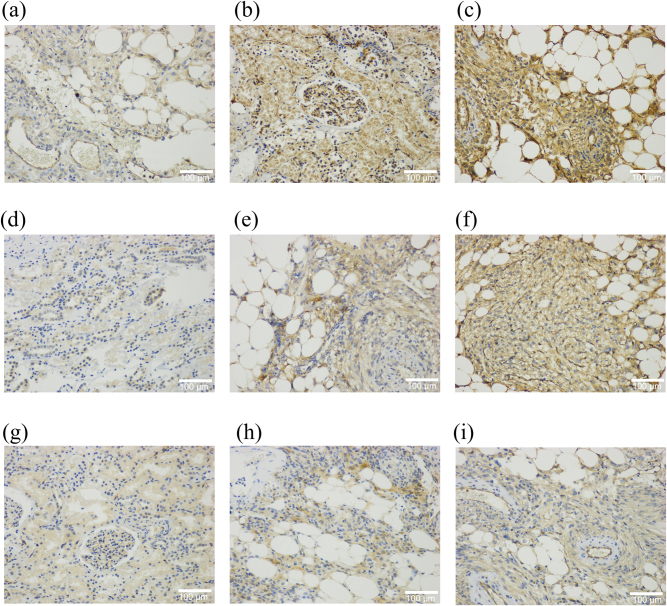
Immunohistochemical expression of *CD34*, *PPARG*, and *PTEN* in normal kidney tissues, non-hemorrhagic AMLs, and hemorrhagic AMLs. Representative immunohistochemical staining images showing the differential expression of *CD34* (a–c), *PPARG* (d–f), and *PTEN* (g–i) among normal renal parenchyma, AMLs without hemorrhage, and hemorrhagic AMLs due to rupture. (a, d, g): normal kidney tissues exhibiting weak or absent *CD34* and *PPARG* staining with preserved *PTEN* expression. (b, e, h): non-hemorrhagic AMLs showing moderate *CD34* and *PPARG* positivity and partial reduction of *PTEN*. (c, f, i): hemorrhagic AMLs demonstrating strong and diffuse *CD34* and *PPARG* expression with marked loss of *PTEN*.

To investigate the molecular mechanisms underlying the high-risk features identified in the clinical model, we evaluated protein expression in 80 AML tissues and 40 normal renal tissues. Using the IRS scoring system, AML specimens exhibited significantly higher immunoreactivity for *CD34* and *PPARG*, and lower immunoreactivity for *PTEN* compared to normal tissues (p<0.05). Crucially, when stratified by rupture status, hemorrhagic AML tissues showed a distinct molecular profile compared to non-hemorrhagic AMLs. Specifically, hemorrhagic tumors exhibited significantly higher rates of high *CD34* and *PPARG* expression (*CD34*: 70.0 % vs. 50.0 %; *PPARG*: 85.0 % vs. 62.5 %) and lower rates of *PTEN* expression (50.0 % vs. 62.5 %) (all p<0.05) (Figure 6 and Table 5). These immunophenotypic patterns suggest that enhanced vascular endothelial activation (*CD34*), metabolic reprogramming (*PPARG*), and loss of tumor suppression (*PTEN*) may drive the vascular fragility observed in high-risk AMLs.

**Table 5:** Immunohistochemical expression of *CD34*, *PPARG*, and *PTEN* in human normal kidney tissues, AML without hemorrhage, and hemorrhagic AMLs due to rupture.

Type	n	Marker	Score 0	Score 1	Score 2	Score 3	p-Value
Normal kidney	40	*CD34*	27	8	5	0	
AML without hemorrhage	40	*CD34*	20	12	6	2	<0.05
AML with hemorrhage	40	*CD34*	12	8	12	8	<0.05
Normal kidney	40	*PPARG*	30	9	1	0	
AML without hemorrhage	40	*PPARG*	15	13	6	6	<0.05
AML with hemorrhage	40	*PPARG*	6	10	14	10	<0.05
Normal kidney	40	*PTEN*	10	15	10	5	
AML without hemorrhage	40	*PTEN*	15	16	8	1	<0.05
AML with hemorrhage	40	*PTEN*	20	10	6	4	<0.05

### Transcriptional and translational validation (qPCR and ELISA)

Consistent with the IHC findings, qPCR analysis confirmed that *CD34* and *PPARG* mRNA levels were significantly upregulated, while *PTEN* mRNA was downregulated in AML tissues compared to normal controls (all p<0.001). In the subgroup analysis, hemorrhagic AML samples exhibited further upregulation of *CD34* and *PPARG* and downregulation of *PTEN* compared with non-hemorrhagic AMLs (p<0.001) (Table 6). ELISA analysis of serum protein concentrations corroborated these tissue-based findings. Circulating levels of *CD34* and *PPARG* were significantly elevated, whereas *PTEN* levels were decreased in the hemorrhagic group compared to the non-hemorrhagic group (p<0.001) (Table 7). These multi-level data provide robust evidence linking the *CD34*/*PPARG*/*PTEN* axis to the pathophysiology of AML rupture.

**Table 6:** Differential mRNA expression of *CD34*, *PPARG*, and *PTEN* in renal angiomyolipoma and normal kidney tissues detected by qPCR.

Gene	Normal (n=40)	AML non-hemorrhagic (n=40)	AML hemorrhagic (n=40)	ANOVA p-Value
*CD34*	1.00 ± 0.40	1.80 ± 0.70	3.20 ± 0.90	p<0.001
*PPARG*	1.20 ± 0.50	2.60 ± 0.90	4.10 ± 1.20	p<0.001
*PTEN*	1.30 ± 0.40	0.90 ± 0.30	0.60 ± 0.20	p<0.001

**Table 7:** Differential protein expression of *CD34*, *PPARG*, and *PTEN* in renal angiomyolipoma and normal kidney tissues detected by ELISA.

Protein	Normal (n=40)	AML non-hemorrhagic (n=40)	AML hemorrhagic (n=40)	ANOVA p-Value
*CD34*	22 ± 8	30 ± 9	45 ± 12	p<0.001
*PPARG*	6 ± 2	11 ± 4	18 ± 5	p<0.001
*PTEN*	6.0 ± 1.3	4.5 ± 1.1	3.2 ± 0.9	p<0.001

## Discussion

### Clinical prediction and molecular insights in renal angiomyolipoma rupture

In the present study, we found that an integrated clinical nomogram incorporating TSC status, tumor size, vascularity, and growth pattern can accurately predict the risk of hemorrhagic rupture in AML, and that this clinical risk is driven by underlying molecular alterations in *CD34*, *PPARG*, and *PTEN* expression. Although AML is the most prevalent benign renal tumor, it poses significant clinical challenges due to the unpredictable risk of spontaneous hemorrhage [28]. Hemorrhagic rupture can lead to hypovolemic shock and mortality, underscoring the critical need for accurate risk stratification [29]. By adopting a two-phase approach, we established this clinically applicable nomogram based on preoperative characteristics and investigated the molecular correlates driving vascular fragility. Importantly, we provide molecular evidence that aberrant angiogenic and metabolic signaling parallels these clinical risk factors, offering a biological rationale for the proposed prediction model [30].

### Clinical determinants of hemorrhage and model robustness

**Figure 7:**
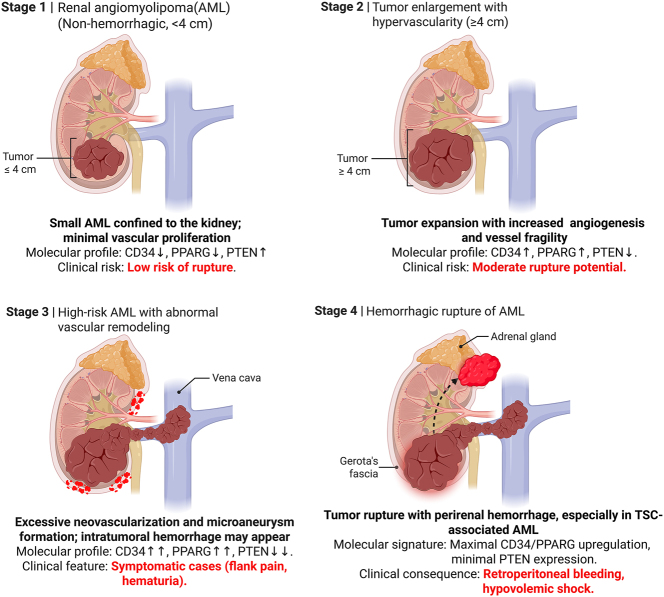
Proposed schematic model illustrating the progressive stages of AML development and hemorrhagic rupture. The figure summarizes the proposed pathophysiological progression of AML from early non-hemorrhagic lesions to advanced hemorrhagic rupture. Stage 1: small AMLs (<4 cm) are confined to the kidney with minimal vascular proliferation, showing low rupture risk (*CD34*↓, *PPARG*↓, *PTEN*↑). Stage 2: tumor enlargement (≥4 cm) is associated with hypervascularity and fragile vasculature, indicating a moderate rupture potential (*CD34*↑, *PPARG*↑, *PTEN*↓). Stage 3: high-risk AMLs exhibit excessive neovascularization and microaneurysm formation, with possible intratumoral hemorrhage and symptomatic presentation (flank pain, hematuria) (*CD34*↑, *PPARG*↑↑, *PTEN*↓↓). Stage 4: tumor rupture occurs, often accompanied by perirenal bleeding and hypovolemic shock, particularly in TSC-associated AML (maximal *CD34*/*PPARG* upregulation and minimal *PTEN* expression).

Tumor size has long been recognized as a primary determinant of hemorrhage, with lesions ≥4 cm generally considered the threshold for intervention [31]. Our results reinforce this, yet the occurrence of rupture in smaller tumors in our cohort highlights the inadequacy of size as a sole predictor [32]. The inclusion of TSC status and tumor growth pattern (exophytic vs. endophytic) significantly improved the model’s discrimination. TSC-associated AMLs often exhibit accelerated growth kinetics and multifocality (Figure 7), biologically predisposing them to instability. Crucially, tumor vascularity emerged as a potent predictor. While previous studies have noted this qualitatively, our nomogram quantifies its contribution [28]. To ensure the reliability of these predictors, we employed an independent temporal validation strategy. Unlike internal resampling methods (*e.g*., bootstrapping) which test stability on the same dataset, our validation cohort was recruited from a distinct time period (2017–2021). The excellent discrimination (AUC=0.967) and calibration observed in this unseen dataset suggest that the model is robust against overfitting and possesses strong generalizability [29]. Although formal Decision Curve Analysis (DCA) was limited by data archival constraints, the high agreement between predicted probability and observed outcome implies a significant clinical net benefit, potentially reducing unnecessary overtreatment while capturing high-risk patients [33].

### Molecular correlates: from clinical phenotype to biological mechanism

A distinct strength of this study lies in bridging the clinical phenotype of “rich vascularity” with its potential underlying molecular machinery involving *CD34*, *PPARG*, and *PTEN* [34]. It is important to distinguish that while our nomogram relies on preoperative clinical variables to ensure practical utility, these molecular markers-typically obtained post-operatively-serve to validate the biological plausibility of the identified risk factors [35]. Specifically, the observed upregulation of *CD34* and *PPARG* and downregulation of *PTEN* in hemorrhagic tissues provide a molecular rationale for vessel rupture. *CD34* overexpression reflects immature, chaotic neovascularization, which is structurally fragile. Similarly, the loss of *PTEN*, a key negative regulator of the PI3K/AKT pathway, may lead to unchecked endothelial proliferation and vascular remodeling [36].

Notably, the mTOR signaling pathway may serve as a critical integration hub linking these clinical and molecular features. TSC1/TSC2 mutations, which define the TSC-AML subtype, lead to the constitutive activation of mTORC1, a master regulator of protein synthesis and cell growth [37]. Our findings of increased *CD34* expression in high-risk AMLs are consistent with the known role of mTOR in promoting hypervascularity through the upregulation of angiogenic factors. Furthermore, *PTE*N acts as a negative regulator of the PI3K/Akt/mTOR axis; thus, the observed loss of *PTEN* expression in our hemorrhagic cohort likely further disinhibits mTOR signaling, exacerbating vascular instability and tumor growth [38]. The concurrent dysregulation of *PPARG* may also intersect with this network, as mTOR signaling has been shown to modulate lipid metabolism and inflammatory responses through *PPARG*-dependent pathways [39]. Therefore, we propose that the TSC-PTEN-mTOR-*PPARG* axis represents a key mechanistic driver of the vascular remodeling and fragility that predispose AML to hemorrhagic rupture.

Regarding the nature of these associations, a critical consideration is whether these molecular alterations are drivers of rupture or secondary consequences of hemorrhage-induced inflammation [37]. As this is a retrospective study, establishing a definitive causal relationship remains challenging. However, we hypothesize that the dysregulation of *CD34*, *PPARG*, and *PTEN* expression likely represents an intrinsic event that predisposes the tumor to rupture [38]. This is supported by our observation that similar expression trends-albeit less pronounced-were present in unruptured AMLs with rich blood supply compared to those with poor blood supply. Nonetheless, it is plausible that once a hemorrhagic event occurs, the subsequent acute inflammatory response and local tissue stress may further exacerbate these molecular aberrations, creating a feedback loop that increases vascular fragility [39]. Therefore, these markers may act both as primary drivers of instability and as secondary mediators that are amplified by the rupture event itself. This observation suggests that dysregulation of the *CD34*-*PPARG*-*PTEN* axis is an intrinsic event promoting vascular instability, rather than solely a downstream effect of bleeding [28].

### Clinical implications, limitations and future directions

The integration of a robust clinical nomogram with mechanistic insights offers a comprehensive framework for AML management. Clinically, the nomogram aids in decision-making for asymptomatic patients: a patient with a small but high-scoring tumor (*e.g*., small size but TSC-positive and hypervascular) might warrant earlier intervention (embolization or nephron-sparing surgery) rather than active surveillance [39]. Biologically, the identification of the *CD34*/*PPARG*/*PTEN* axis suggests potential therapeutic avenues. For instance, pharmacologic modulation of *PPARG* or PI3K/AKT inhibitors (targeting the *PTEN* pathway) could theoretically stabilize tumor vasculature [40]. Future research should focus on prospective, multicenter trials to validate the nomogram’s utility in diverse populations.

**Figure 8:**
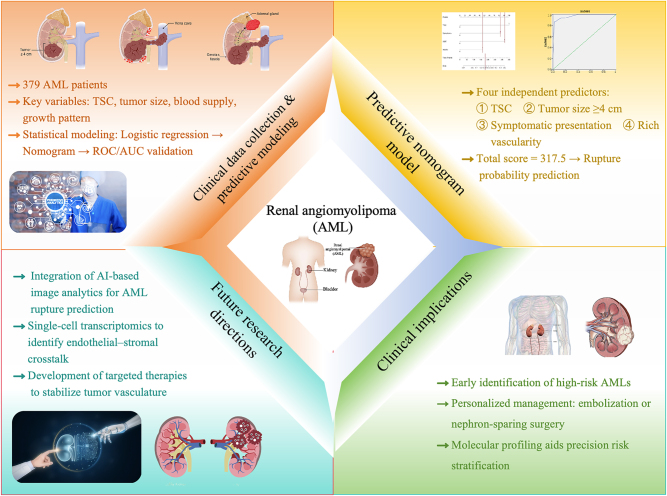
Integrated conceptual framework of AML rupture risk prediction and translational perspectives. The schematic summarizes the overall framework of the present study, integrating clinical data analysis, predictive modeling, clinical translation, and future research perspectives in AML. The study enrolled 379 AML patients, with key predictive variables including tuberous sclerosis complex (TSC), tumor size, tumor vascularity, and growth pattern. Logistic regression analysis was used to construct a predictive nomogram, which was validated by ROC/AUC analyses to estimate rupture probability. The resulting model identified four independent predictors: (1) TSC, (2) tumor size ≥4 cm, (3) symptomatic presentation, and (4) rich vascularity, allowing for individualized rupture risk assessment. Clinical implications include early identification of high-risk AMLs, personalized management strategies (*e.g.*, embolization or nephron-sparing surgery), and molecular profiling-based precision risk stratification. Future directions emphasize the integration of AI-based imaging analytics, single-cell transcriptomic mapping of endothelial-stromal interactions, and the development of molecularly targeted therapies to stabilize tumor vasculature and prevent rupture.

Additionally, mechanistic studies using *in vitro* or animal models are needed to definitively prove the causal role of these molecules in vascular rupture. Furthermore, the potential involvement of local inflammatory cell infiltration, such as macrophages, in the rupture process or as a secondary tissue response warrants thorough investigation. As illustrated in Figure 8, future research utilizing single-cell transcriptomics will be instrumental in identifying the specific crosstalk between these inflammatory cells and the vascular-stromal microenvironment, potentially uncovering new therapeutic targets to stabilize fragile AML vessels [41].

Several limitations must be acknowledged. First, the retrospective nature of the study introduces inherent selection bias. Second, regarding the assessment of clinical utility, we acknowledge the value of DCA. However, due to the retrospective nature of the study spanning over a decade and specific limitations in archiving raw probability data for the entire cohort, we were unable to retrospectively generate the specific data points required for a new DCA plot at this stage. For the same archiving reason, we were unable to generate visual calibration plots or calculate Brier scores, relying instead on the Hosmer-Lemeshow test for calibration assessment. Nevertheless, the excellent calibration (Hosmer-Lemeshow p=0.561) and discrimination (AUC=0.967) observed in the independent validation cohort strongly imply that clinical decisions based on this nomogram would yield net benefits [42]. Third, immunohistochemical scoring, despite being performed by two independent pathologists with consensus resolution, remains semi-quantitative [43]. Finally, while we propose the *CD34*-*PPARG*-*PTEN* axis as a driver of hemorrhage, prospective molecular profiling of biopsy specimens prior to rupture would be required to definitively establish causality [44].

In summary, this study establishes a validated, clinically practical nomogram for the pre-operative prediction of AML hemorrhagic rupture, supported by independent internal temporal validation [43]. Furthermore, we elucidated that the clinical risk of hemorrhage is mirrored by a distinct molecular profile characterized by high *CD34*/*PPARG* and low *PTEN* expression. This “clinical-molecular” coherence not only validates the biological basis of our predictive model but also highlights potential biomarkers and therapeutic targets for preventing life-threatening tumor rupture [44].
